# Associations between health-related quality of life and subsequent need for specialized palliative care and hospital utilization in patients with gastrointestinal cancer—a prospective single-center cohort study

**DOI:** 10.1007/s00520-024-08509-z

**Published:** 2024-04-27

**Authors:** Stine Gerhardt, Kirstine Skov Benthien, Suzanne Herling, Bonna Leerhøy, Lene Jarlbaek, Peter-Martin Krarup

**Affiliations:** 1 Digestive Disease Center, Copenhagen University Hospital - Bispebjerg, Bispebjerg Bakke 23, 2400 Copenhagen, NV Denmark; 2 Palliative Care Unit, Copenhagen University Hospital - Hvidovre, Hvidovre, Denmark; 3 REHPA - Danish Knowledge Centre for Rehabilitation and Palliative Care, University of Southern Denmark, Nyborg, Denmark; 4 Centre for Translational Research, Copenhagen University Hospital - Bispebjerg, Copenhagen, Denmark; 5 The Neuroscience Center, University of Copenhagen, Rigshospitalet, Copenhagen, Denmark

**Keywords:** Palliative care, Health-related quality of life, Neoplasms, Patient-reported outcome measures

## Abstract

**Background:**

We lack knowledge of which factors are associated with the risk of developing complex palliative care needs. The aim of this study was to investigate the associations between patient-reported health-related quality of life and subsequent referral to specialized palliative care (SPC) and hospital utilization.

**Methods:**

This was a prospective single-center cohort study. Data on patient-reported outcomes were collected through the European Organization of Research and Treatment of Cancer Questionnaire-Core-15-Palliative Care (EORTC QLQ-C15-PAL) at the time of diagnosis. Covariates and hospital utilization outcomes were collected from medical records. Adjusted logistic and Poisson regression were applied in the analyses. Participants were newly diagnosed with incurable gastrointestinal cancer and affiliated with a palliative care case management intervention established in a gastroenterology department.

**Results:**

Out of 397 patients with incurable gastrointestinal cancer, 170 were included in the study. Patients newly diagnosed with incurable gastrointestinal cancer experienced a substantial burden of symptoms. Pain was significantly associated with subsequent referral to SPC (OR 1.015; 95% CI 1.001–1.029). Patients with lower education levels (OR 0.210; 95% CI 0.056–0.778) and a Charlson Comorbidity Index score of 2 or more (OR 0.173; 95% CI 0.041–0.733) were less likely to be referred to SPC. Pain (IRR 1.011; 95% CI 1.005–1.018), constipation (IRR 1.009; 95% CI 1.004–1.015), and impaired overall quality of life (IRR 0.991; 95% CI 0.983–0.999) were significantly associated with increased risk of hospital admissions.

**Conclusion:**

The study indicates a need for interventions in hospital departments to identify and manage the substantial symptom burden experienced by patients, provide palliative care, and ensure timely referral to SPC.

## Background

The referral of patients with incurable cancer to specialized palliative care (SPC) is challenged by a lack of strategies for referral practice, inequality in age, diagnoses, and region, as well as physician attitudes [1–3].

Patients with incurable gastrointestinal cancer have a substantial symptom burden escalating towards the end of life [4]. In cross-sectional studies, symptoms such as pain, fatigue, and constipation are associated with hospitalizations and reduced health-related quality of life (HRQoL) [4–12]. This often demands either generalist palliative care (PC), which is provided by healthcare professionals who integrate PC into their clinical practice without specialized training, such as those in surgical departments, or specialized PC delivered by PC specialists addressing patients’ complex needs from dedicated teams in hospices or specialized PC hospital units [13]. Due to limited capacity, less than 50% of patients with incurable cancer are provided SPC in Denmark, and 23% of patients referred to SPC do not proceed to an SPC unit [14]. They either die before admittance, are not admitted due to a shortage of resources in SPC, or fail to meet the referral criteria [15]. The median survival time from referral to SPC in Denmark is 36 days, emphasizing the importance of timely referral [14]. Visitation criteria for SPC include complex palliative symptomatology, a life-threatening illness, and a patient’s awareness of the prognosis of the disease [16]. However, initiation of SPC can mistakenly be perceived as a marker of end-stage disease, although disease stage alone does not determine the complexity of palliative care (PC) needs to be required for visitation [17, 18].

SPC has previously been associated with improved HRQoL [19–22]. Other advantages include decreased hospital admissions and shorter hospital stays [23]. Evidence of effective generalist PC interventions is yet to be established [24]. The associations between patient-reported HRQoL, demographic characteristics, and the referral to SPC have previously been studied with attention to the time point of admission to SPC [25–27]. However, there is an information gap concerning the risk factors associated with developing complex PC needs in patients not already affiliated with SPC.

Hospital departments outside of SPC play a crucial role in identifying and managing patients needing PC. At the Digestive Disease Center, Copenhagen University Hospital—Bispebjerg, Denmark, a nurse-led palliative care case management intervention for gastrointestinal cancer (PalMaGiC) was initiated in 2013 to optimize the deliverance of PC. Knowing the characteristics of patients who are at risk of needing SPC despite well-organized PC interventions provided by hospital departments not specialized in palliative care may, in the future, improve timely and relevant referral of patients to SPC [5, 8, 12].

The aim of this study was to investigate the associations between patient-reported symptoms, problems, and HRQoL with subsequent referral to SPC and hospital utilization among patients with newly diagnosed incurable gastrointestinal cancer.

## Methods

### Design

In this prospective single-center cohort study, we explored factors associated with future contact with SPC and hospital utilization.

### Participants and settings

Patients from the Digestive Disease Center, Copenhagen University Hospital—Bispebjerg, affiliated with the PalMaGiC from December 2018 to May 2022, were included. PalMaGiC is a case management intervention established in the Digestive Disease Center, a gastroenterology hospital department, and developed to improve early identification and management of palliative care needs and quality of life. PalMaGiC receives approximately 120 patients with incurable gastrointestinal cancers annually and is led by two specialist nurses performing symptom assessment, care planning, care coordination, and needs-based follow-up. The PalMaGiC intervention is described in detail in another publication [28].

The recruitment took place at the first contact with the PalMaGiC nurse. This contact was established shortly after the patient’s cancer disease was recognized as incurable. Patients were consecutively included in the study and asked to provide demographic information and complete the European Organization of Research and Treatment of Cancer QoL Questionnaire Core-15-Palliative care (EORTC QLQ-C15-PAL) questionnaire, which is a shortened version of European Organization of Research and Treatment of Cancer QoL Questionnaire Core-30 (EORTC QLQ-C30) and includes issues important to patients in palliative care, at the initial interview (baseline) [29]. This interview took place either at the Digestive Disease Center or by telephone. Data on diagnosis, treatment decisions, antineoplastic treatment, comorbidity, and healthcare utilization were collected from the patient’s medical records. Data were prospectively entered into a database.

Inclusion criteria: patients ≥ 18 years old with incurable cancers of the esophagus, stomach, pancreas, bile duct, colon, or rectum not being treated with curative intent.

Exclusion criteria: not speaking and understanding Danish well enough or unable to complete the questionnaire due to cognitive inability or functional deterioration, and concurrent affiliation with SPC at the start of PalMaGiC or referral immediately after.

### Variables

#### Outcomes

The primary outcome was a referral to SPC (yes/no). Secondary outcomes were the total number of hospital admissions, defined as at least one overnight stay, and length of visit, defined as the total of days at the hospital. Data were true to time and collected from patient records.

#### Independent variables

Baseline patient-reported symptoms, physical function, emotional function, and overall QoL were measured on EORTC QLQ-C15-PAL. EORTC QLQ-C15-PAL is a patient-reported outcome measure (PROM) validated in Danish and contains 15 items, encompassing two multi‐item functional scales (emotional and physical), two multi‐item symptom scales (fatigue and pain,) a five single‐item symptoms (nausea/vomiting, dyspnea, insomnia, appetite loss, and constipation), and an overall QoL question. All items are rated on a scale of 1 (not at all) to 4 (very much), except for the overall QoL question, which is rated from 1 (very poor) to 7 (excellent). All scores are transformed to a 0–100-scale range: a higher score indicates better physical and emotional functioning and overall QoL, while a higher score on symptom scales indicates an increased symptom burden. The questionnaire covers an assessment of the past week [29].

#### Covariates

Age, sex, cancer site, metastases (locally advanced or metastases), and educational level (master’s level or above/primary bachelor’s level) were included as covariates. Furthermore, we included comorbidity as the calculated score according to the Charlson Comorbidity Index (CCI) based on the International Classification of Disease (ICD-10) and categorized into 0 = normal, 1 = moderate, and ≥ 2 = severe [30, 31].

Patients’ diagnoses were grouped into five areas of gastrointestinal cancers: “ECS” (esophagus, cardia, stomach); “pancreas”; “colorectal” (colon, rectum, and appendix); “bile ducts” (cholangiocarcinoma and gall bladder), and “other” (hepatocellular carcinoma, small intestine and unknown).

### Analyses

Descriptive statistics, including frequencies, medians, and ranges, were applied for patient characteristics, information on SPC, symptoms, and overall QoL. Crude and adjusted logistic regression were used to investigate the associations between symptoms, physical function, emotional function, and overall QoL and the binary outcome referral to specialized palliative care (yes/no). The associations between symptoms, physical function, emotional function, overall QoL and hospital stays, and the total length of stay were analyzed using Poisson regression. An offset variable was created to adjust for follow-up time. A model was created for each of the EORTC QLQ-C15-PAL subscales: symptoms, physical function, emotional function, and overall QoL as a continuous variable. Patients were followed from the first interview with the PalMaGiC-nurse (baseline) and censored at transfer to SPC, death, or end of study (September 2022).

Sensitivity analyses were performed for variables with statistically significant association to the outcomes by dichotomizing at two different cut-off points reflecting if the patient “experienced” the symptom/problem scores of ≥ 33 (a little) and experienced the symptom/problem “severely” with scores of ≥ 66 (quite a bit and very much) [32]*.* All adjusted analyses were adjusted for age, sex, cancer site, metastases, comorbidity, physical function, pain, and overall QoL. In the sensitivity analyses, testing “experienced pain” and “severe pain” as independent variables, we excluded adjusting pain in the model to avoid collinearity.

Finally, missingness in QoL data was investigated using patient characteristics and information on referral to SPC from medical files for patients not included due to lack of baseline information about HRQoL. Included and excluded patients were compared using the Wilcoxon rank sum test for continuous variables and the chi-squared test for categorical variables, respectively. Data on educational level were not available for excluded patients. The statistical analyses were performed in SAS 9.4. A *P* value of < 0.05 was considered statistically significant.

## Results

### Study sample

**Fig. 1:**
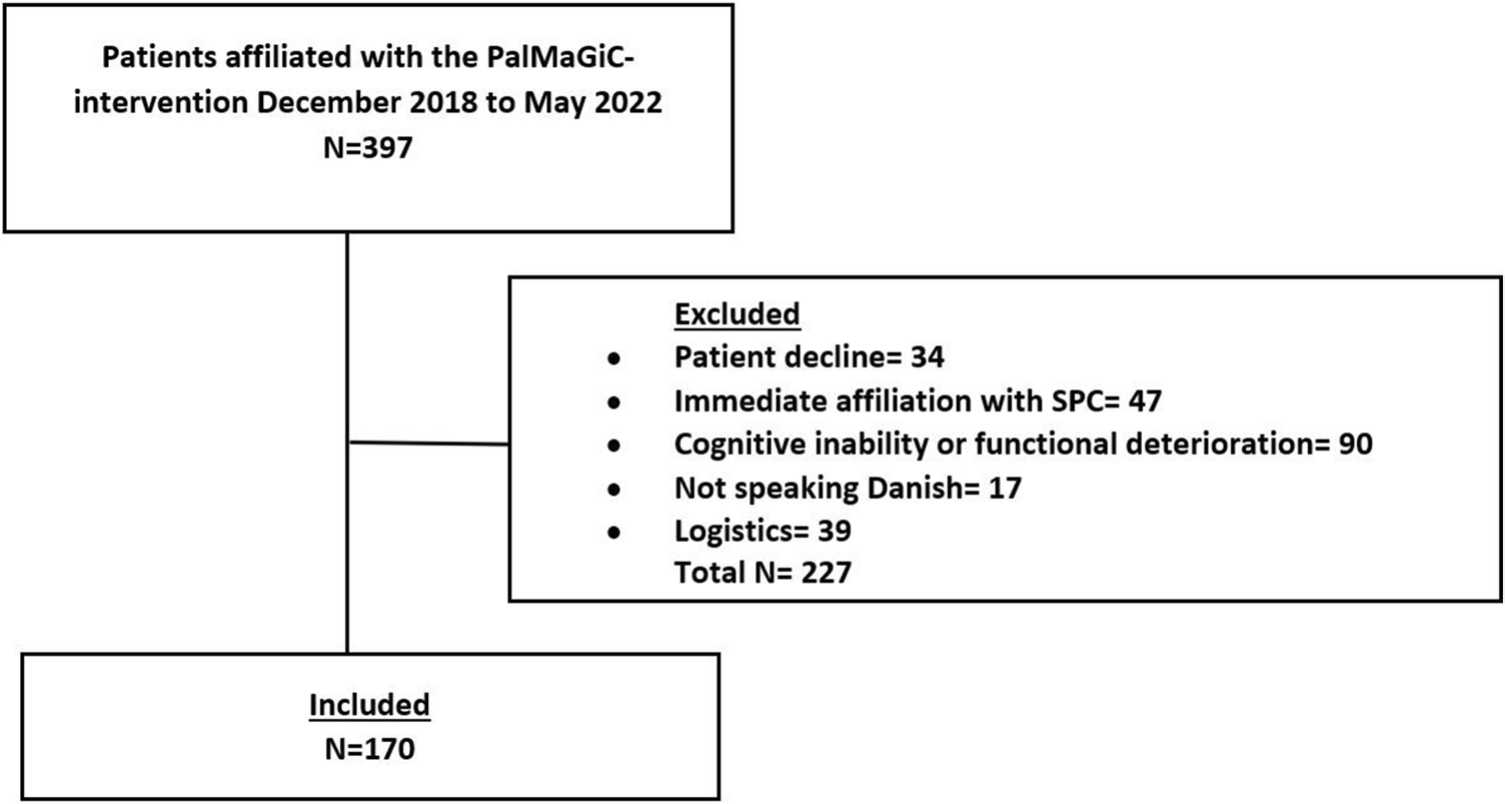
Inclusion flow chart

From December 2018 to May 2022, 397 patients were referred to PalMaGiC. After excluding 227, 170 patients were included in the study (Fig. 1). Fifty-five percent were men, and the median age was 74 (IQR 66–81). At the end of the study period, 92% of the patients had died. Follow-up time was a median of 69 (IQR 28–199) days. Days from PalMaGiC to death were median 114 (IQR 47–189.5) for ECS, 76 (IQR 40–173) for pancreatic, 65 (IQR 45–138) for bile duct, and 87 (IQR 41–317) for colorectal cancers. Characteristics of included and excluded patients are summarized in Table 1.

**Table 1:** Baseline characteristics for included and excluded patients in the study cohort

N = 397	Included *n* = 170	Excluded *n* = 227	*P* value*
Age, median (IQR)	74 (66–81)	78 (67–86)	0.004
Sex, *n* (%)			
Male	93 (55%)	124 (55%)	1.000
Days from incurable cancer diagnosis to baseline, median (IQR)	9 (3–29)	14.5 (4–87)	0.021
Days of follow-up time, median (IQR)	69 (28–199)	42 (17–137)	0.002
Days from baseline to death, median (IQR)	85 (41–210)	70 (24–189)	0.042
Cancer site, *n* (%)			0.565
Esophagus, cardia, stomach (ECS)	32 (19%)	48 (21%)	
Pancreas	50 (29%)	51 (22%)	
Bile ducts	17 (10%)	23 (10%)	
Colon/rectum	62 (36%)	88 (39%)	
Other	9 (5%)	17 (8%)	
Charlson Comorbidity Index, *n* (%)			0.106
0	105 (62%)	116 (51%)	
1	51 (30%)	83 (37%)	
2 +	14 (8%)	27 (12%)	
Disease, *n* (%)			0.035
Locally advanced	41 (24%)	77 (34%)	
Metastases	129 (76%)	150 (66%)	
Antineoplastic treatment, *n* (%)			
Chemotherapy	78 (46%)	74 (33%)	0.009
Radiation	31 (18%)	38 (17%)	0.893
Immunotherapy	8 (5%)	7 (3%)	0.595
Education, *n* (%)			
Master’s level or above	34 (20%)	-	-
Primary-bachelor’s level	136 (80%)	-	-
Cohabitation status, *n* (%)			0.011
Living alone	86 (51%)	149 (66%)	
Living with spouse/partner	72 (42%)	67 (30%)	
Other	12 (7%)	11 (4%)	

*P value for performed Wilcoxon rank sum test and chi-squared test for comparison of included vs excluded patients. Charlson Comorbidity Index scores of 0 = normal, 1 = moderate, ≥ 2 = severe

### Baseline HRQoL

The symptoms with the highest proportion of patients with scores > 33 were fatigue (85%), appetite loss (72%), pain (60%), and sleeplessness (53%). Furthermore, 85% reported impaired overall QoL, 68% reported impaired physical function, and 48% reported impaired emotional function (i.e., scores ≤ 66).

**Fig. 2:**
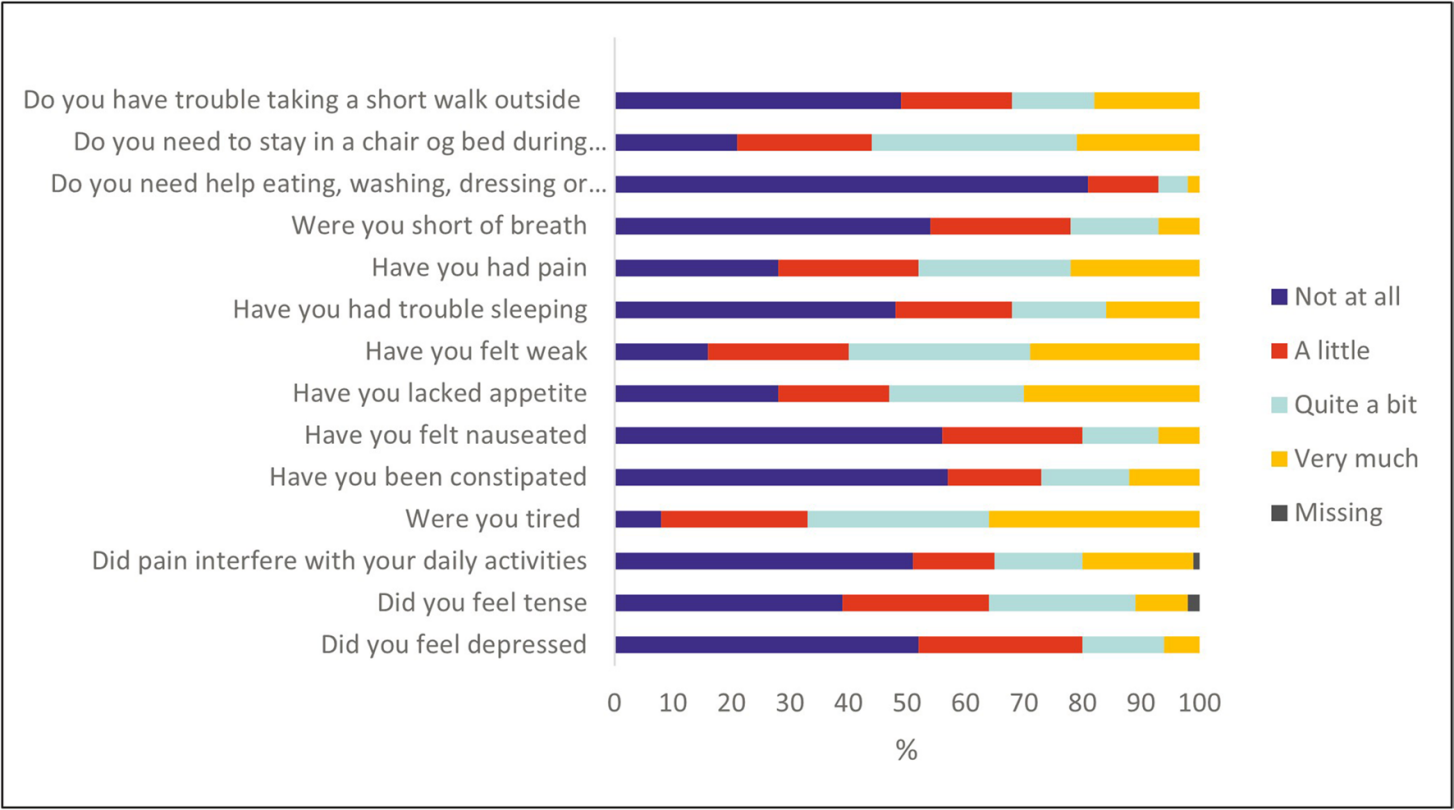
The distribution of the baseline scores of symptoms, physical function, and emotional function on EORTC QLQ-C15-PAL by patients (%)

The score reported for overall QoL was a mean score of 45 (SD 26.67). The physical function score was 53 (SD 26.90), and the emotional function score was 72 (SD 26.13). The highest mean symptom scores were fatigue (a mean score of 63, SD 30.30), appetite loss (mean 52, SD 39.56), and pain (mean 41, SD 35.35). The remaining mean symptom scores at baseline were sleeplessness 34 (SD 38.03), constipation 27 (SD 36.33), dyspnea 25 (SD 31.93), and nausea 17 (SD 28.19). The distribution of questionnaire responses is shown in Fig. 2.

### Referral to specialized palliative care (SPC)

Of the included patients, 74% were referred to SPC, and 86% of these patients were admitted to SPC, while 18 patients died before admittance to an SPC unit. The number of days from the start of PalMaGiC to transfer to SPC was 52 (IQR 21–133). The number of days from referral to transfer to SPC was a median of 7 (IQR 3–13), and the median number of days from transfer to death was 30 (IQR 8–70) in this sample. Overall, 58% of the excluded patients were affiliated with SPC either immediately after the incurable diagnosis (*n* = 47) or referred later (*n* = 85), which was significantly fewer than included patients referred to SPC (*p* = 0.002).

### The association between baseline HRQoL and referral to SPC

Pain was significantly associated with subsequent referral to SPC (OR 1.015; 95% CI 1.001–1.029). No other symptoms, physical function, emotional function, or overall QoL were associated with subsequent referral to SPC (Table 2). In the sensitivity analysis, severe pain of ≥ 66 was significantly associated with referral to SPC (OR 2.885; 95% CI 1.035–8.056), but not pain ≥ 33. Patients with a lower level of education (OR 0.210; 95% CI 0.056–0.778), a CCI of 2 or more (OR 0.173; 95% CI 0.041–0.733), and a locally advanced cancer (compared to metastatic disease) (OR 0.279; 95% CI 0.111–0.696) were less likely to be referred to SPC.

**Table 2:** Factors associated with subsequent need for specialized palliative care

*N* = 170	Crude			Adjusted		
Referral to specialized palliative care	OR	95% CI	*P* value	OR	95% CI	*P* value
Age						
≤ 70	1.534	0.733–3.211	0.256	1.228	0.508–2.969	0.648
Sex						
Male	0.508	0.249–1.036	0.062	1.853	0.796–4.311	0.156
Diagnosis group						
Colorectal	Ref	Ref	Ref	Ref	Ref	Ref
ECS	0.557	0.217–1.428	0.502	0.781	0.263–2.322	0.968
Pancreatic	0.924	0.383–2.228	0.423	0.912	0.333–2.500	0.656
Bile duct	0.948	0.266–3.372	0.552	1.049	0.196–5.615	0.636
Other	0.364	0.086–1.543	0.237	0.357	0.065–1.948	0.253
Education						
Master’s level or above	Ref	Ref	Ref	Ref	Ref	Ref
Primary-bachelor’s level	**0.309**	**0.102**–**0.933**	**0.037**	**0.210**	**0.056**–**0.778**	**0.037**
Charlson Comorbidity Index						
0	Ref	Ref	Ref	Ref	Ref	Ref
1	0.775	0.353–1.700	0.056	1.073	0.424–2.715	0.069
2 +	**0.106**	**0.030**–**0.371**	**0.007**	**0.173**	**0.041–0.733**	**0.012**
Disease						
Metastases	Ref	Ref	Ref	Ref	Ref	Ref
Locally advanced	**0.252**	**0.119**–**0.535**	** < 0.001**	**0.279**	**0.111**–**0.696**	**0.063**
Symptoms						
Physical function	0.998	0.986–1.011	0.811	0.999	0.982–1.017	0.936
Emotional function	1.000	0.987–1.013	0.948	1.004	0.986–1.023	0.650
Nausea	1.005	0.992–1.018	0.465	0.996	0.979–1.014	0.674
Pain	**1.013**	**1.002**–**1.024**	**0.017**	**1.015**	**1.001**–**1.029**	**0.039**
Fatigue	1.010	0.999–1.022	0.077	1.012	0.992–1.031	0.233
Dyspnea	1.003	0.992–1.014	0.567	1.003	0.989–1.018	0.665
Sleeplessness	0.999	0.990–1.008	0.822	0.998	0.987–1.010	0.772
Appetite loss	1.006	0.998–1.015	0.149	1.002	0.990–1.014	0.733
Constipation	1.004	0.994–1.014	0.420	0.998	0.984–1.011	0.711
Overall QoL	0.998	0.985–1.011	0.716	1.005	0.998–1.023	0.570

Crude and adjusted logistic regression adjusted for age, sex, diagnosis group, comorbidity, metastases, education, physical function, pain, and overall quality of life. Charlson Comorbidity Index scores of 0 = normal, 1 = moderate, ≥ 2 = severe. *ECS* esophagus, cardia, stomach. *Ref* reference. *QoL* quality of life

### The associations between baseline HRQoL and hospital admissions and length of stay

Overall, patients had a median of 2 (IQR 1–2) hospital admissions, and the median total length of stay was 9 days (IQR 2–18) in the follow-up period. After adjustment for the covariates, the incidence rate of hospital admissions in patients diagnosed with cancer in the bile ducts was significantly higher than in patients diagnosed with colorectal cancer (IRR 2.443; 95% CI, 1.217–4.906) (Table 3). In addition, pain (IRR 1.011; 95% CI, 1.005–1.018), constipation (IRR 1.009; 95% CI 1.004–1.015), and impaired overall QoL (IRR 0.991; 95% CI 0.983–0.999) at baseline were significantly associated with increased risk of subsequent hospital admissions (Table 4).

**Table 3:** Factors associated with subsequent risk of hospital admissions and length of stay

*N* = 170	Admissions			Length of stay		
	IRR	95% CI	*P* value	IRR	95% CI	*P* value
Age						
≥ 70	0.829	0.559–1.229	0.351	0.958	0.620–1.480	0.846
Sex						
Male	1.401	0.963–2.056	0.078	1.344	0.879–2.054	0.172
Diagnosis group						
Colorectal	Ref	Ref	Ref	Ref	Ref	Ref
ECS	1.008	0.542–1.874	0.980	0.951	0.488–1.853	0.882
Pancreatic	1.362	0.877–2.115	0.170	1.040	0.628–1.723	0.878
Bile duct	**2.443**	**1.217**–**4.906**	**0.012**	**2.678**	**1.296**–**5.534**	**0.008**
Other	0.916	0.326–2.573	0.868	1.235	0.471–3.237	0.668
Education						
Primary-bachelor’s level	Ref	Ref	Ref	Ref	Ref	Ref
Master’s level or above	1.170	0.708–1.935	0.539	0.932	0.552–1.574	0.792
Charlson Comorbidity Index						
0	Ref	Ref	Ref	Ref	Ref	Ref
1	0.895	0.556–1.438	0.645	0.775	0.441–1.362	0.376
2 +	0.394	0.152–1.018	0.054	0.542	0.221–1.328	0.180
Disease						
Metastases	Ref	Ref	Ref	Ref	Ref	Ref
Locally advanced	1.420	0.865–2.332	0.166	1.678	0.997–2.854	0.056

Poisson adjusted regression of hospital admissions and length of stay by covariates adjusted for age, sex, diagnosis group, comorbidity, metastases, education, physical function, pain, and overall quality of life

**Table 4:** Health-related quality of life associated with subsequent hospital admissions and length of stay

*N* = 170	Crude			Adjusted		
Admissions and length of stay	*IRR*	*95% CI*	*P value*	*IRR*	*95% CI*	*P value*
Physical function						
Admissions	**0.982**	**0.974–0.989**	** < 0.0001**	0.995	0.985**–**1.004	0.254
Length of stay	**0.986**	**0.978–0.994**	**0.001**	1.000	0.990**–**1.011	0.929
Emotional function						
Admissions	**0.988**	**0.980–0.996**	**0.003**	1.006	0.996**–**1.015	0.251
Length of stay	**0.988**	**0.980–0.996**	**0.005**	1.005	0.995**–**1.016	0.333
Nausea						
Admissions	**1.016**	**1.001–1.021**	** < 0.0001**	1.006	0.998**–**1.015	0.143
Length of stay	**1.013**	**1.006–1.021**	**0.001**	1.005	0.995**–**1.015	0.347
Pain						
Admissions	**1.015**	**1.009–1.020**	** < 0.0001**	**1.011**	**1.005–1.018**	**0.001**
Length of stay	**1.012**	**1.001–1.018**	** < 0.0001**	**1.088**	**1.009–1.016**	**0.014**
Fatigue						
Admissions	**1.015**	**1.009–1.022**	** < 0.0001**	0.998	0.987**–**1.008	0.667
Length of stay	**1.016**	**1.009–1.023**	** < 0.0001**	0.999	0.988**–**1.012	0.985
Dyspnea						
Admissions	**1.008**	**1.001–1.014**	**0.023**	1.005	0.998**–**1.011	0.166
Length of stay	1.004	0.997**–**1.012	0.283	1.002	0.994**–**1.009	0.680
Sleeplessness						
Admissions	**1.008**	**1.003–1.013**	**0.004**	1.002	0.996**–**1.008	0.547
Length of stay	1.004	0.998**–**1.010	0.171	0.997	0.990**–**1.004	0.351
Constipation						
Admissions	**1.011**	**1.006–1.017**	** < 0.0001**	**1.009**	**1.004–1.015**	**0.001**
Length of stay	**1.012**	**1.007–1.018**	** < 0.0001**	**1.009**	**1.004–1.015**	**0.001**
Appetite loss						
Admissions	**1.011**	**1.006–1.016**	** < 0.0001**	1.004	0.999**–**1.010	0.127
Length of stay	**1.014**	**1.008–1.019**	** < 0.0001**	**1.008**	**1.002–1.014**	**0.015**
Overall QoL						
Admissions	**0.985**	**0.979–0.991**	** < 0.0001**	**0.991**	**0.983–0.999**	**0.030**
Length of stay	**0.983**	**0.976–0.989**	** < 0.0001**	**0.986**	**0.978–0.995**	**0.002**

Hospital admissions and length of stay by symptoms, problems, and health-related quality of life. Crude and adjusted Poisson regression adjusted for age, sex, diagnosis group, comorbidity, metastases, education, physical function, severe pain, and overall Quality of Life. Bold indicates statistically significant results

Bile duct cancer (IRR 2.678; 95% CI, 1.296–5.534), pain (IRR 1.088; 95% CI 1.002–1.016), appetite loss (IRR 1.008; 95% CI 1.002–1.014), constipation (IRR 1.009; 95% CI 1.004–1.015) and impaired overall QoL (IRR 0.986; 95% CI 0.978–0.995) at baseline were associated with increased length of stay (Table 4).

## Discussion

### Symptom burden and referral to SPC

This study found that patients newly diagnosed with incurable gastrointestinal cancer experienced a substantial burden of symptoms, including pain, impaired physical function, impaired emotional function, and impaired overall QoL, which make them candidates for an increased focus on PC. Previous studies have identified similar symptom burden by focusing on symptoms on admittance to SPC or at initiation of antineoplastic treatment [4, 8, 26, 33]. In the present study, baseline pain was associated with subsequent referral to SPC, which may be considered a proxy for palliative care needs. In a large Danish nationwide register-based study of patients with esophageal, stomach, colorectal, and pancreatic cancers, the frequency of pain was 77%, with mean scores between 53 and 58 at the time of admittance to SPC [26], thus indicating that pain is a persistent symptom with increasing intensity over time. Moreover, pain in malignant diseases is associated with fear towards the end of life and is thus a complex condition that often requires SPC [34–36]. A cross-sectional observational study evaluating the thresholds for clinical importance of EORTC QLQ-C15-PAL by Pilz et al. included patients receiving PC. The patients who reported clinically important symptoms had lower mean scores for the physical and emotional function and higher scores for symptoms than the scores found in our study, with the exception of physical function scores [32]. Therefore, healthcare professionals must include the patient in the interpretation by discussing the impact of the symptom on their everyday life and the subsequent decision of treatment and referral to SPC.

Interestingly, no other symptoms or problems were associated with the subsequent need for SPC in this study. A possible explanation could be the fluctuation of symptoms throughout the trajectory or later escalation for some patients. Another explanation may be the increased focus on PC needs by the PalMaGiC intervention. This is the first study to investigate the associations between baseline HRQoL and subsequent SPC needs. Other studies investigated systematic screening to trigger referral to SPC. These studies used standardized referral criteria based on expert consensus, such as the National Comprehensive Cancer Network’s palliative care screening items (NCCN) [37], consisting of screening for uncontrolled symptoms, functional status, prognosis, and distress. The authors found increased SPC consultations when standardized referral criteria were systematically assessed [38–40].

The use of PROM in the identification of patients in need of SPC is challenged by the many patients unable to complete the questionnaire due to deterioration in the last phase of life [41–43]. This was confirmed in the present study, where a large proportion of patients did not complete the questionnaire. Using PROM to identify patients in need of SPC should not favor questionnaire completers and neglect non-completers who are in poorer condition and possibly more burdened by symptoms. The White Paper on Outcome Measures in Palliative Care recommends using proxy assessments provided by healthcare professionals or caregivers for patients unable to complete questionnaires to accommodate this common issue [44]. Although the evidence of accuracy is inconsistent, this could offer valuable information on the patient’s symptoms and prompt clinical awareness of the need for SPC [44, 45]. A clinician-reported outcome measure, such as the proxy-reported Integrated Palliative care Outcome Scale (IPOS) staff version, can accommodate this issue [46]. IPOS staff version assesses patients’ symptoms facilitated by healthcare professionals through questions covering how the patient has been affected by the symptom during the past week with five response categories from “Not at all” to “Overwhelming,” including “cannot assess.” Studies have shown the IPOS staff version to be valid and reliable [46, 47].

### Hospital utilization

Patients reporting pain, constipation, and impaired overall QoL at the time of a recognized incurable gastrointestinal cancer were more likely to be hospitalized later in the trajectory and to spend more days in the hospital. Baseline appetite loss was associated with longer hospital stays. Nipp et al. reported similar findings by hospitalized patients with advanced cancers upon admission [8]. Physical symptoms were associated with longer duration of hospital stay and more frequent unplanned readmissions, confirming the findings in the present prospective study that patient-reported symptoms at the time of diagnosis of incurable cancer can result in hospitalizations later in the disease trajectory [8]. Seventer et al. also found that a higher symptom score and lower physical function score were associated with a higher risk of hospital admissions in patients with advanced gastrointestinal cancers [48]. Symptoms of constipation, including pain and appetite loss, reported by patients who had more hospital admissions and longer hospital stays, can be caused by malignant bowel obstruction [49]. This may explain the higher risk of hospital admissions and longer stays in the hospital in patients reporting baseline constipation in this study. Malignant bowel obstruction is highly prevalent in patients with gastrointestinal cancers due to carcinomatosis or intestinal tumor blockage [49, 50]. The diagnosis requires careful assessment and a clinical strategy different from that used to treat constipation [6, 49].

### What this study adds

This study demonstrates that despite a substantial burden of symptoms, pain at the time of the diagnosis in patients with incurable gastrointestinal cancer was the only factor associated with subsequent referral to SPC. This can be hypothesized to be related to providing a palliative care intervention (PalMaGiC). However, the substantial symptoms increase the risk of hospital admissions and days in the hospital. Thus, it is essential that hospital departments focus on providing generalist palliative care, including adequate identification and management of symptoms. We suggest that baseline pain should lead to immediate initiation of pain management and a follow-up assessment of symptoms shortly after.

Unsuccessful pain management on follow-up assessment should prompt healthcare professionals to refer patients to SPC to offer the best possible pain relief to improve QoL.

The short prognosis of the subgroup of patients with bile duct cancers and their high risk of hospital utilization should prompt referral to SPC shortly after the incurable diagnosis is confirmed and if they present with baseline pain.

Finally, we must intensify the assessment and treatment of the subgroup of patients with baseline constipation, with attention to the possibility of the differential diagnosis of malignant bowel obstruction.

Future research should address the symptom burden in the time gap between baseline assessment and referral to SPC or death, including the frequency of follow-up PROM assessments. Additionally, future investigation should focus on proxy assessment of symptoms and developing more adequate referral criteria based on the characteristics of patients with gastrointestinal cancers, including customized criteria for the group of patients unable to complete PROMs.

### Strength and limitations

A strength of this study is the prospectively collected PROM data from patients with gastrointestinal cancer with a limited lifespan, which described the symptom burden at the start of the trajectory in a gastroenterology hospital department.

A limitation is the selection bias reflected in the high proportion of excluded patients, possibly severely burdened by symptoms. However, we were able to analyze baseline characteristics from these excluded patients and provide a complete description of our cohort.

Another limitation is the way education level has been grouped together. Specifically, the grouping of bachelor’s education with primary and secondary education levels may obscure the actual impact of education on the outcomes being studied. This grouping may have resulted in underestimating the associations with higher education. A limitation is also the relatively small sample and single-center setting in the study, compromising the generalizability of the study due to the diversity of organizations of generalist palliative care in hospital departments in Denmark and internationally.

Finally, we are limited in categorizing cancer sites in upper and lower gastrointestinal cancers. Upper gastrointestinal cancers differ substantially from colorectal cancers in prognosis, symptom burden, and hospital utilization rate, potentially leading to missed signals in our statistical analysis.

## Conclusion

Patients in this study presented with a high symptom burden early in their disease trajectory, highlighting the need for interventions in general hospital departments to identify and manage symptoms and provide palliative care besides ensuring timely referral to SPC.

## Data Availability

No datasets were generated or analyzed during the current study.

## References

[1] Hui D, Meng YC, Bruera S, Geng Y, Hutchins R, Mori M (2016). Referral criteria for outpatient palliative cancer care: a systematic review. The oncologist (Dayton, Ohio).

[2] Adsersen M, Thygesen LC, Neergaard MA, Jensen AB, Sjogren P, Damkier A (2017). Admittance to specialized palliative care (SPC) of patients with an assessed need: a study from the Danish palliative care database (DPD). Acta Oncol.

[3] Kirsten W, Monika KK, Nadia S, Gary MR, Lisa WL, Camilla Z (2012). Referral practices of oncologists to specialized palliative care. J Clin Oncol.

[4] Merchant SJ, Brogly SB, Booth CM, Goldie C, Nanji S, Patel SV (2019). Palliative care and symptom burden in the last year of life: a population-based study of patients with gastrointestinal cancer. Ann Surg Oncol.

[5] Okafor PN, Stobaugh DJ, Nnadi AK, Talwalkar JA (2017). Determinants of palliative care utilization among patients hospitalized with metastatic gastrointestinal malignancies. Am J Hosp Palliat Med.

[6] Paul Olson TJ, Pinkerton C, Brasel KJ, Schwarze ML (2014). Palliative surgery for malignant bowel obstruction from carcinomatosis: a systematic review. JAMA Surg.

[7] Teunissen SC, Wesker W, Kruitwagen C, de Haes HC, Voest EE, de Graeff A (2007). Symptom prevalence in patients with incurable cancer: a systematic review. J Pain Symptom Manage.

[8] Nipp RD, El-Jawahri A, Moran SM, D'Arpino SM, Johnson PC, Lage DE (2017). The relationship between physical and psychological symptoms and health care utilization in hospitalized patients with advanced cancer. Cancer.

[9] Prigerson HG, Bao Y, Shah MA, Paulk ME, LeBlanc TW, Schneider BJ (2015). Chemotherapy use, performance status, and quality of life at the end of life. JAMA Oncol.

[10] Earle CC, Landrum MB, Souza JM, Neville BA, Weeks JC, Ayanian JZ (2008). Aggressiveness of cancer care near the end of life: is it a quality-of-care issue?. J Clin Oncol.

[11] Earle CC, Park ER, Lai B, Weeks JC, Ayanian JZ, Block S (2003). Identifying potential indicators of the quality of end-of-life cancer care from administrative data. J Clin Oncol.

[12] Hui D, Kim SH, Roquemore J, Dev R, Chisholm G, Bruera E (2014). Impact of timing and setting of palliative care referral on quality of end-of-life care in cancer patients. Cancer.

[13] Danish Health Authorities. Recommendations for Palliative Care (2017) https://www.sst.dk/da/sygdom-ogbehandling/~/media/79CB83AB4DF74C80837BAAAD55347D0D.ashx

[14] Danish Multidisciplinary Cancer Group-Palliative care DMCG-PAL. Danish Palliative Database. http://www.dmcgpal.dk/373/dansk-palliativ-database-(dpd) 2021.

[15] The National Audit Office: Report on Access to Specialized Palliative Care (2020) https://rigsrevisionen.dk/Media/637830383760074804/SR1819.pdf

[16] The Danish National Board of Health. Recommendations of Palliative Care. https://www.sst.dk/da/sygdom-og-behandling/~/media/79CB83AB4DF74C80837BAAAD55347D0D.ashx.

[17] Adolfsson K, Kreicbergs U, Bratthäll C, Holmberg E, Björk-Eriksson T, Stenmarker M (2022). Referral of patients with cancer to palliative care: attitudes, practices and work-related experiences among Swedish physicians. Eur J Cancer Care..

[18] Wentlandt K, Krzyzanowska MK, Swami N, Rodin GM, Le LW, Zimmermann C (2012). Referral practices of oncologists to specialized palliative care. J Clin Oncol.

[19] Nottelmann L, Groenvold M, Vejlgaard TB, Petersen MA, Jensen LH (2017). A parallel-group randomized clinical trial of individually tailored, multidisciplinary, palliative rehabilitation for patients with newly diagnosed advanced cancer: the Pal-Rehab study protocol. BMC Cancer.

[20] Zimmermann C, Swami N, Krzyzanowska M, Hannon B, Leighl N, Oza A (2014). Early palliative care for patients with advanced cancer: a cluster-randomised controlled trial. Lancet (London, England).

[21] Kavalieratos D, Corbelli J, Zhang D, Dionne-Odom JN, Ernecoff NC, Hanmer J (2016). Association between palliative care and patient and caregiver outcomes: a systematic review and meta-analysis. JAMA.

[22] Haun MW, Estel S, Rucker G, Friederich HC, Villalobos M, Thomas M, et al (2017) Early palliative care for adults with advanced cancer. The Cochrane database of systematic reviews 6:Cd011129. 10.1002/14651858.CD011129.pub2. 10.1002/14651858.CD011129.pub2 PMC6481832 28603881

[23] Schnipper LE, Smith TJ, Raghavan D, Blayney DW, Ganz PA, Mulvey TM (2012). American Society of Clinical Oncology identifies five key opportunities to improve care and reduce costs: the top five list for oncology. J Clin Oncol.

[24] Ernecoff NC, Check D, Bannon M, Hanson LC, Dionne-Odom JN, Corbelli J (2020). Comparing specialty and primary palliative care interventions: analysis of a systematic review. J Palliat Med.

[25] Hansen MB, Nylandsted LR, Petersen MA, Adsersen M, Rojas-Concha L, Groenvold M (2020). Patient-reported symptoms and problems at admission to specialized palliative care improved survival prediction in 30,969 cancer patients: a nationwide register-based study. Palliat Med.

[26] Hansen MB, Ross L, Petersen MA, Groenvold M (2019). Age, cancer site and gender associations with symptoms and problems in specialised palliative care: a large, nationwide, register-based study. BMJ Support Palliat Care.

[27] Wadhwa D, Popovic G, Pope A, Swami N, Le LW, Zimmermann C (2018). Factors associated with early referral to palliative care in outpatients with advanced cancer. J Palliat Med.

[28] Gerhardt S, Leerhøy B, Jarlbaek L, Herling S (2023). Qualitative evaluation of a palliative care case management intervention for patients with incurable gastrointestinal cancer (PalMaGiC) in a hospital department. Eur J Oncol Nurs.

[29] Groenvold M, Petersen MA, Aaronson NK, Arraras JI, Blazeby JM, Bottomley A (2006). The development of the EORTC QLQ-C15-PAL: a shortened questionnaire for cancer patients in palliative care. Eur J Cancer.

[30] Charlson ME, Pompei P, Ales KL, MacKenzie CR (1987). A new method of classifying prognostic comorbidity in longitudinal studies: development and validation. J Chronic Dis.

[31] Thygesen SK, Christiansen CF, Christensen S, Lash TL, Sørensen HT (2011). The predictive value of ICD-10 diagnostic coding used to assess Charlson comorbidity index conditions in the population-based Danish National Registry of Patients. BMC Med Res Methodol.

[32] Pilz MJ, Aaronson NK, Arraras JI, Caocci G, Efficace F, Groenvold M (2020). Evaluating the thresholds for clinical importance of the EORTC QLQ-C15-PAL in patients receiving palliative treatment. J Palliat Med.

[33] Dalhammar K, Kristensson J, Falkenback D, Rasmussen BH, Malmström M. Symptoms, problems and quality of life in patients newly diagnosed with oesophageal and gastric cancer - a comparative study of treatment strategy. BMC cancer. 2022;22(1):434-. 10.1186/s12885-022-09536-x. 10.1186/s12885-022-09536-x PMC9022327 35448961

[34] Sholjakova M, Durnev V, Kartalov A, Kuzmanovska B (2018). Pain relief as an integral part of the palliative care. Open Access Maced J Med Sci.

[35] Kroenke K, Theobald D, Wu J, Loza JK, Carpenter JS, Tu W (2010). The association of depression and pain with health-related quality of life, disability, and health care use in cancer patients. J Pain Symptom Manage.

[36] Steinhauser KE, Christakis NA, Clipp EC, McNeilly M, McIntyre L, Tulsky JA (2000). Factors considered important at the end of life by patients, family, physicians, and other care providers. JAMA.

[37] Levy MH, Back A, Benedetti C, Billings JA, Block S, Boston B (2009). NCCN clinical practice guidelines in oncology: palliative care. J Natl Compr Canc Netw.

[38] Glare P, Plakovic K, Schloms A, Egan B, Epstein AS, Kelsen D (2013). Study using the NCCN guidelines for palliative care to screen patients for palliative care needs and referral to palliative care specialists. J Natl Compr Canc Netw.

[39] Adelson K, Paris J, Horton JR, Hernandez-Tellez L, Ricks D, Morrison RS (2017). Standardized criteria for palliative care consultation on a solid tumor oncology service reduces downstream health care use. J Oncol Pract.

[40] Churchill I, Turner K, Duliban C, Pullar V, Priestley A, Postma K (2020). The use of a palliative care screening tool to improve referrals to palliative care services in community-based hospitals: a quality improvement initiative. J Hosp Palliat Nurs.

[41] Graupner C, Kimman ML, Mul S, Slok AHM, Claessens D, Kleijnen J (2021). Patient outcomes, patient experiences and process indicators associated with the routine use of patient-reported outcome measures (PROMs) in cancer care: a systematic review. Support Care Cancer.

[42] Müller E, Mayer-Steinacker R, Gencer D, Keßler J, Alt-Epping B, Schönsteiner S (2023). Feasibility, use and benefits of patient-reported outcome measures in palliative care units: a multicentre observational study. BMC palliative care.

[43] Tavares APdS, Paparelli C, Kishimoto CS, Cortizo SA, Ebina K, Braz MS (2017). Implementing a patient-centred outcome measure in daily routine in a specialist palliative care inpatient hospital unit: An observational study. Palliative medicine.

[44] Bausewein C, Daveson BA, Currow DC, Downing J, Deliens L, Radbruch L (2016). EAPC White Paper on outcome measurement in palliative care: improving practice, attaining outcomes and delivering quality services – Recommendations from the European Association for Palliative Care (EAPC) Task Force on Outcome Measurement. Palliat Med.

[45] Oldenburger E, Devlies J, Callens D, De Roo ML (2023). Patient-reported outcomes versus proxy-reported outcomes in supportive and palliative care: a summary of recent literature. Curr Opin Support Palliat Care.

[46] Murtagh FEM, Ramsenthaler C, Firth A, Groeneveld EI, Lovell N, Simon ST (2019). A brief, patient- and proxy-reported outcome measure in advanced illness: validity, reliability and responsiveness of the Integrated Palliative care Outcome Scale (IPOS). Palliat Med.

[47] Sakurai H, Miyashita M, Morita T, Naito AS, Miyamoto S, Otani H (2021). Comparison between patient-reported and clinician-reported outcomes: validation of the Japanese version of the Integrated Palliative care Outcome Scale for staff. Palliat Support Care.

[48] Seventer EE, Fish MG, Fosbenner K, Kanter K, Mojtahed A, Allen JN (2021). Associations of baseline patient-reported outcomes with treatment outcomes in advanced gastrointestinal cancer. Cancer.

[49] Franke AJ, Iqbal A, Starr JS, Nair RM, George JTJ (2017). Management of malignant bowel obstruction associated with GI cancers. Journal of oncology practice.

[50] Grotmol KS, Lie HC, Loge JH, Aass N, Haugen DF, Stone PC (2019). Patients with advanced cancer and depression report a significantly higher symptom burden than non-depressed patients. Palliat Support Care.

